# Acceleration Sensitivity in Bulk-Extensional Mode, Silicon-Based MEMS Oscillators

**DOI:** 10.3390/mi9050233

**Published:** 2018-05-12

**Authors:** Beheshte Khazaeili, Jonathan Gonzales, Reza Abdolvand

**Affiliations:** 1 Department of Electrical and Computer Engineering, University of Central Florida, Orlando, FL 32816, USA; reza@eecs.ucf.edu; 2 School of Electrical and Computer Engineering, Oklahoma State University, Tulsa, OK 74074, USA; Jonathan.gonzales@okstate.edu

**Keywords:** MEMS resonators, acceleration sensitivity, vibration sensitivity, nonlinearity

## Abstract

Acceleration sensitivity in silicon bulk-extensional mode oscillators is studied in this work, and a correlation between the resonator alignment to different crystalline planes of silicon and the observed acceleration sensitivity is established. It is shown that the oscillator sensitivity to the applied vibration is significantly lower when the silicon-based lateral-extensional mode resonator is aligned to the <110> plane compared to when the same resonator is aligned to <100>. A finite element model is developed that is capable of predicting the resonance frequency variation when a distributed load (i.e., acceleration) is applied to the resonator. Using this model, the orientation-dependent nature of acceleration sensitivity is confirmed, and the effect of material nonlinearity on the acceleration sensitivity is also verified. A thin-film piezoelectric-on-substrate platform is chosen for the implementation of resonators. Approximately, one order of magnitude higher acceleration sensitivity is measured for oscillators built with a resonator aligned to the <100> plane versus those with a resonator aligned to the <110> plane (an average of ~5.66 × 10^−8^ (1/g) vs. ~3.66 × 10^−9^ (1/g), respectively, for resonators on a degenerately n-type doped silicon layer).

## 1. Introduction

In recent years, the application of micro-machined, silicon-based resonators in timing has been growing steadily [1,2,3]. Internet of things, mobile and wearable, automotive, and smart infrastructure monitoring are some examples of applications where extremely small and ultra-stable MEMS-based oscillators could play an important role in the system performance. The stability of these oscillators is affected by environmental conditions such as temperature, humidity, pressure, magnetic field, acceleration/vibration, etc. A change in one or more of these environmental conditions can either vary the resonance frequency of the resonator or the oscillator loop phase [4]. In the former case, any change in the resonance frequency of the resonator will directly impact the oscillator frequency, as other electronic components are assumed to be broadband. In the latter case, change in the phase of the oscillator loop is compensated for by a shift in the oscillation frequency, so that the loop phase is maintained at 360°, which is a required condition for oscillation [4].

A significant mechanical vibration could exist in the typical operating environment of the electronics utilized in certain applications such as cell-phone towers, aircrafts, automotives, aerospace vehicles, and radar towers. Such vibration results in frequency instability and performance deterioration of the oscillators onboard. Applied acceleration to the oscillator can induce a change in the resonance frequency of the mechanical resonator, as the stress applied to the resonator through the suspension tethers will change the instantaneous resonance frequency of the device. Alternatively, the stray capacitance and inductance of the circuit could change as the circuit board is deformed due to the applied vibration, resulting in variation of the oscillator loop phase [4].

In order to characterize the shift in resonance frequency of a mechanical resonator due to the applied stress, a parameter defined as a stress-frequency coefficient is typically used. The stress-frequency coefficient is shown to depend on the combination of two factors: the geometrical deformation and the nonlinear elastic properties of the material used in the resonator [5]. In quartz crystal resonators, this coefficient is extensively studied and proven to be dependent on the crystal cut [6,7,8,9,10,11]. Several methods have been used for reducing the vibration sensitivity of quartz-based oscillators. For example, it is suggested that compensation of the acceleration-induced shift in oscillation frequency is possible by applying an appropriately phase-shifted electrical signal to the crystal’s electrodes that is proportional to the mechanical vibration [12]. Active compensation of acceleration sensitivity has also been studied in recent years. In [13,14,15], authors have achieved low vibration-sensitive oscillators through electrical compensation of the oscillator output using sensors that are strategically mounted to accurately measure the applied acceleration. A similar method of electronic vibration-induced noise cancelation has been used for optoelectronic oscillators as well [16]. In a different approach, two crystals with anti-parallel acceleration-sensitivity characteristics have been used in an oscillator circuit so that the net acceleration sensitivity vector can be adjusted to zero [11,17,18]. Similar compensation methods using discrete or stacked crystals are represented in [19]. Choosing an appropriate shape and place for mounting supports in order to reduce stress, and also employing precise fabrication techniques to accurately locate and shape the designed mounting structures, are other important factors considered in previous works [20]. The type of the material used in the oscillator is also another degree of freedom that could be utilized for reducing acceleration sensitivity. As explained in [21], experimental results obtained from two different air-dielectric cavity oscillators made from two different materials, ceramic and aluminum, showed almost one sixth times lower acceleration sensitivity for the one made of ceramic. Optical oscillators also showed low acceleration sensitivities due to their small size [22]. Other interesting improvements are reported in the field of optical cavity resonators. The acceleration sensitivity of a cavity-stabilized laser is decreased significantly using the feedforward correction of acceleration [23]. In addition, recent research on the acceleration sensitivity of optical cavity resonators shows that performance deterioration due to the external mechanical vibration can be minimized by the appropriate design of the cavity structure [24,25,26]. 

As mentioned above, material nonlinearity is an important factor affecting the sensitivity of the resonators to mechanical vibration [4,8,27,28]. Since nonlinear elastic constants in crystalline material are anisotropic, the orientation-dependency of the vibration sensitivity due to this nonlinearity is potentially predictable. It has been shown that the stress-frequency coefficient of stress-compensated (SC)-cut quartz is nearly zero. Therefore, the cuts near SC-cut quartz are known to exhibit enhanced performance metrics in terms of force-frequency, resonance amplitude-frequency, acceleration-frequency, intermodulation, and dynamic thermal-frequency stability [5,29,30]. 

The oscillator market has recently begun to accept silicon-based MEMS resonators as an alternative for the long-stablished quartz resonators [31,32]. In theory, MEMS resonators should exhibit lower sensitivity to acceleration compared with quartz resonators because of their smaller size and mass. It is of great importance to study the physics of vibration sensitivity in silicon-based resonators in order to understand the limits and to develop proper design guidelines to reduce vibration induced instability in silicon-MEMS oscillators. Several factors such as shape, position, and number of suspension tethers, aspect ratio of the resonator dimensions, device layer thickness, and mode shape can affect the acceleration sensitivity of a MEMS resonator. Different studies have been performed to analyze the acceleration sensitivity of these resonators. In [33], the acceleration sensitivity of a lame-mode resonator supported with four anchors located at four corners of the square-shape structure is studied. In this work, it is shown that adding an extra anchor at the center of the resonator is effective at reducing the acceleration sensitivity. The acceleration sensitivity of a small-gap capacitive MEMS oscillator is studied in [34]. In capacitive resonators, the vibration-induced variation of the gap size and the transducer capacitance overlap area results in nonlinear changes in electrostatic stiffness, which results in a shift in resonance frequency. 

This paper focuses on the acceleration sensitivity of silicon-based MEMS resonators. It is shown that the acceleration sensitivity of silicon-based resonators is orientation-dependent and has a correlation with the elastic properties of silicon including the Poisson ratio and nonlinear elastic coefficients. A finite element model in COMSOL is developed that predicts the hypothesized orientation-dependent acceleration sensitivity, and presented experimental results agree with the hypothesis as well. Recently, the preliminary results suggesting the orientation-dependency of the vibration sensitivity for silicon-based MEMS resonators was presented by the authors [35]. This work is an extension of the work that confirms the earlier results with a novel finite element model (FEM) and an observation of the same trends on more resonator samples.

This paper is organized as follows. The theory of acceleration sensitivity is presented in Section 2. In Section 3, the resonators used in this work are introduced and characterized. The finite element model for simulating acceleration sensitivity is explained in Section 4. Experimental results for both nonlinearity and vibration sensitivity measurements are demonstrated in Section 5. Finally, the study is summed up in Section 6.

## 2. Theory of Acceleration Sensitivity

Acceleration sensitivity can be defined as frequency instability of a resonator due to the mechanical vibration introduced by the environment. Applied acceleration will act on the resonator mass to produce a force, and it consequently induces stress and strain within the resonant body. There are two main causes of vibration sensitivity: geometric nonlinearity and material nonlinearity. If the applied strain due to the acceleration is large enough, the device dimensions/geometry will change (i.e., geometric nonlinearity) and consequently the resonance frequency will be affected. In addition, if the elastic stiffness coefficients of the material are strain-dependent (i.e., material elastic nonlinearity), the effective stiffness of the resonator changes due to the applied vibration and the natural resonance frequency will change as a result.

The change in the natural resonance frequency of device as a function of the applied acceleration can be represented as follows [8]:

(1) $$f\left(\vec{a}\right)=f_{0}\left(1+\vec{\Gamma }\cdot \vec{a}\right)$$

in which *f*_0_ is the natural resonance frequency when there is no acceleration, $\vec{\Gamma }$ is the acceleration sensitivity vector, and $\vec{a}$ is the applied acceleration in a certain direction.

Using Equation (1), $\vec{\Gamma }$ can be found by

(2) $$\vec{\Gamma }\cdot \vec{a}=\Delta f/f_{0}$$

in which ∆*f* is the shift in resonance frequency due to the applied vibration. If the applied acceleration is a constant DC vibration, a constant shift will happen at resonance frequency. However, a sinusoidal acceleration will result in a frequency modulation, and the resonance frequency will change periodically from *f*_0_ − ∆*f* to *f*_0_ + ∆*f* at a rate equal to the vibration frequency. Since ∆*f* is normally a very small value, it is not easily detectable. Hence, to measure acceleration sensitivity, the resonator should be employed in an oscillator circuit. Once the output power spectrum of the oscillator is analyzed, the sidebands that appear at an offset equal to the vibration frequency of the carrier power are detectable. Let us assume the output voltage of the oscillator is given by

(3) $$V\left(t\right)=V_{0}\cos\left(\phi \left(t\right)\right)$$

in which *ϕ*(*t*) is the phase of the oscillator, which can be found by integrating the frequency

(4) $$\phi \left(t\right)=2\pi \int_{t_{0}}^{t}f\left(t^{\prime }\right)dt^{\prime }$$

in which *f(t)* is the frequency of the oscillator under applied vibration. Considering a sinusoidal acceleration Equation (5), resonance frequency will be modified as shown in Equation (6)

(5) $$\vec{a}=\vec{A}\cos\left(2\pi f_{v}t\right)$$

(6) $$f\left(\vec{a}\right)=f_{0}\left(1+\vec{\Gamma }\cdot \vec{A}\cos\left(2\pi f_{v}t\right)\right)$$

Therefore, the phase is calculated as

(7) $$\phi \left(t\right)=2\pi f_{0}t+\left(\frac{\Delta f}{f_{v}}\right)\sin\left(2\pi f_{v}t\right),\;\Delta f=f_{0}\left(\vec{\Gamma }\cdot \vec{A}\right)$$

By substituting Equation (7) in Equation (3) the output voltage of the oscillator is calculated as follows:

(8) $$V\left(t\right)=V_{0}\cos\left(2\pi f_{0}t+\left(\frac{\Delta f}{f_{v}}\right)\sin\left(2\pi f_{v}t\right)\right)$$

As seen in Equation (8), the output of the oscillator under a sinusoidal vibration is a frequency-modulated signal that can be expanded using a series of Bessel functions as follows:

(9) $$V\left(t\right)=V_{0}\left[J_{0}\left(\beta \right)\cos\left(2\pi f_{0}t\right)+J_{1}\left(\beta \right)\cos\left(2\pi \left(f_{0}+f_{v}\right)t\right)+J_{1}\left(\beta \right)\cos\left(2\pi \left(f_{0}-f_{v}\right)t\right)+\ldots \right]$$

in which *β* = ∆*f*/*f_v_* = $\left(\vec{\Gamma }\cdot \vec{A}\right)$ *f*_0_/*f_v_* is the modulation index. For *β* < 0.1, *J*_0_*(β) =* 1*, J*_1_*(β) = β/*2, and *J_n_(β) =* 0 for *n >* 2*.* Hence, for small modulation indices, the output spectrum of the oscillator only contains the main resonance frequency and two sidelobes at *f*_0_ ± *f*_v_. As seen from Equation (9), the magnitude of these sidelobes (*β/*2) depends on the acceleration amplitude and frequency, resonance frequency of the resonator, and the acceleration sensitivity vector. The ratio of the power in these two sidelobes to the power of the carrier is derived through the following equation

(10) $$L_{v}={\left(\frac{J_{1}\left(\beta \right)}{J_{0}\left(\beta \right)}\right)}^{2}$$

or equivalently in decibels notation

(11) $$L_{v,dB}=20\log\left(\frac{J_{1}\left(\beta \right)}{J_{0}\left(\beta \right)}\right)=20\log\left(\frac{\beta }{2}\right)=20\log\left(\frac{\left(\vec{\Gamma }\cdot \vec{A}\right)f_{0}}{2f_{v}}\right)$$

So, acceleration sensitivity in *i*th direction, $\vec{\Gamma }$, can be found as follows:

(12) $$\Gamma_{i}=\left(2f_{v}/A_{i}f_{0}\right)10^{L_{v}/20}$$

in which *f_v_* is the vibration frequency, *A_i_* is the amplitude of vibration in *i*th direction, and *L_v_* is the difference between the carrier signal power and sidebands power in *dB*.

The amplitude of total acceleration sensitivity along *x*, *y,* and *z* direction is calculated as follows:

(13) $$\Gamma_{t}=\sqrt{\Gamma_{x}{}^{2}+\Gamma_{y}{}^{2}+\Gamma_{z}{}^{2}}$$

## 3. Resonator Design and Characterization

**Figure 1:**
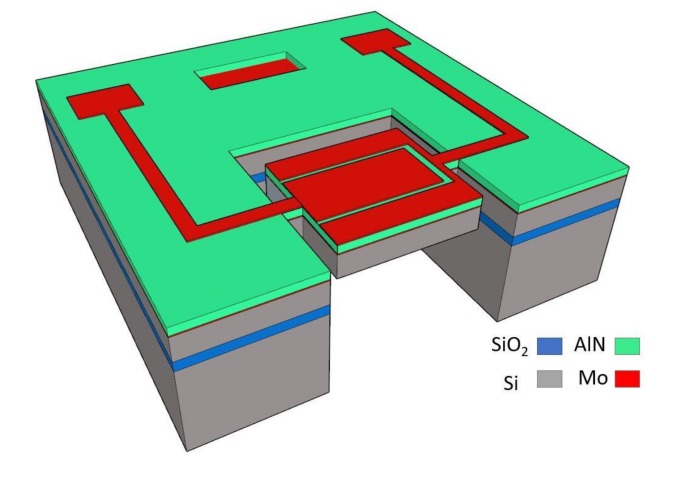
The schematic of a typical TPoS resonator used in this work.

Thin-film piezoelectric-on-substrate (TPoS) platform is chosen in this work for prototyping the silicon-based resonators as they offer high quality factor and low insertion loss [36]. The schematic of a typical TPoS resonator used in this work is shown in Figure 1, in which a thin sputtered piezoelectric AlN layer, sandwiched between top and bottom electrodes made of Molybdenum, is stacked on top of a <100> silicon layer. The top electrode is patterned on the resonant structure to enable two-port operation of the resonator. The specific interdigitated configuration could be used to excite higher harmonic lateral-extensional resonance modes [37,38]. However, in this work the first harmonic resonance is used in the oscillator, and the top electrodes are connected to each other for the resonator to be operated in a one-port configuration. In order to study the effect of orientation on acceleration sensitivity, resonators with identical design are rotated to align to different crystalline orientations. The stack layers of the resonators used in this work include 8 μm silicon, 1 μm AlN, and 100 nm Molybdenum used as electrodes. The substrate for all resonators is a phosphorus-doped silicon-on-insulator (SOI) wafer with a device layer doped at a concentration of ~5 × 10^19^ cm^−3^.

**Figure 2:**
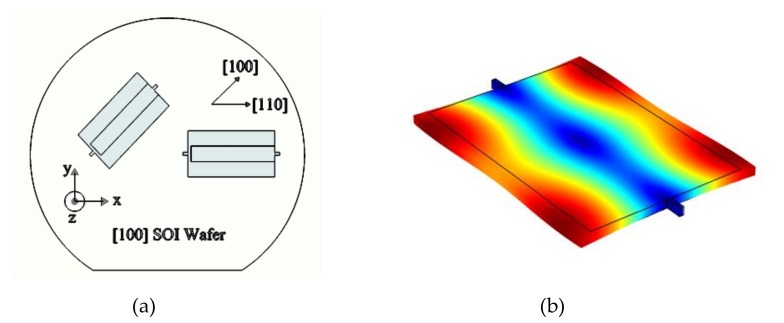
(**a**) A schematic representation of the relative position of <100> and <110> resonators on the [100] wafer and (**b**) the simulated lateral extensional mode-shape of a resonator used in this work.

A schematic representing the relative position of the resonators aligned to <100> and <110> orientations on the [100] SOI wafer is shown in Figure 2. The fundamental lateral extensional mode-shape of the resonant structure used in this work is also shown in this figure.

**Figure 3:**
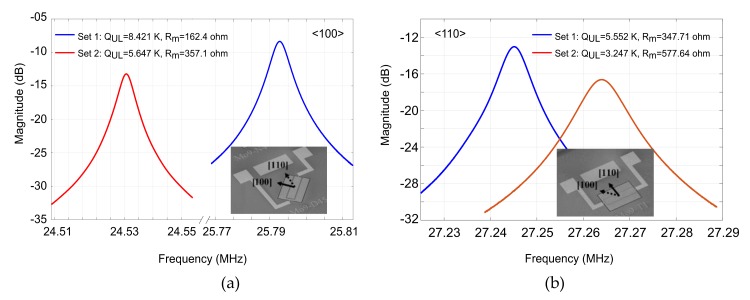
The frequency response (i.e., S21) and the SEM of the two sets of resonators used in this work aligned to <100> (**a**) and <110> (**b**) silicon planes.

The frequency response (S21 amplitude) and the scanning electron micrograph (SEM) of the resonators used in this study are also shown in Figure 3.

## 4. Finite Element Simulation

A theoretical or simulation model that could be used to predict and improve acceleration sensitivity of resonators is very desirable for design purposes. Due to the nonlinear nature of vibration sensitivity, developing such tools is not straightforward. Complicated equations have been developed for vibration sensitivity of bulk mode resonators, indicating dependency of vibration sensitivity to a complex function of linear and nonlinear elastic constants [39]. However, a finite element model can be more useful, as numerical solutions have recently found popularity due to the great progress made in computational speed and capacity.

In order to develop a finite element model for this purpose, geometric and material nonlinearities should be both included. Geometric nonlinearity is already predefined in commercial FEM software packages such as COMSOL, but capturing material nonlinearity of silicon is more challenging.

### 4.1. Geometric Nonlinearity

When there is relatively large deformation and rotation, the approximations typically used in linear elastic equations break apart, and it is necessary to identify the distinction between deformed and undeformed configuration, and the nonlinear definitions of stress and strain should be utilized as opposed to the simplified engineering definitions.

The definition of linear engineering stain is shown in Equation (14), which needs to be replaced by Equation (15), the nonlinear Green–Lagrangian strain, in case of having rotation or large deformation.

(14) $$e_{X}=\frac{\partial u}{\partial X}$$

(15) $$S_{X}=\frac{\partial u}{\partial X}+\frac{1}{2}{\left(\frac{\partial u}{\partial X}\right)}^{2}+\frac{1}{2}{\left(\frac{\partial V}{\partial X}\right)}^{2}+\frac{1}{2}{\left(\frac{\partial w}{\partial X}\right)}^{2}$$

Therefore, it is required to modify the equations of the FEM simulator in order to consider nonlinear definitions of strain. COMSOL software will provide the option to consider geometric nonlinearity in the model, and, once selected, the Green–Lagrangian strain will be utilized in all calculations.

### 4.2. Material Nonlinearity

In general, there is a nonlinear relationship between stress and strain for any arbitrary material. Normally, for small strain values, one could use a linear approximate instead of the nonlinear relation. However, for large values of strain, the nonlinear equation should be considered. In the case of simulating acceleration sensitivity, we are dealing with small values of resonance frequency shift, in the range of ppb. Thus, considering nonlinear terms is critical in accurately predicting very small changes in resonance frequency, even though the applied acceleration and the resulted strain are small. Equation (16) shows the nonlinear relationship between second Piola–Kirchhoff (2nd PK) stress and Green–Lagrangian strain for silicon [40,41]

(16) $$T_{ij}\left(X\right)=C_{ijkl}S_{kl}+\frac{1}{2}C_{ijklmn}S_{kl}S_{mn}$$

in which *C_ijkl_*, *C_ijklmn_*, *T_ij_*, and *S_kl_* are linear and nonlinear elastic stiffness coefficients, 2nd PK stress, and Green–Lagrangian strain, respectively. In order to capture acceleration sensitivity of the resonators correctly, we propose to define this nonlinear stress-strain relation in COMSOL as follows. First, Equation (16) will be rewritten as

(17) $$T_{ij}\left(X\right)=(C_{ijkl}+\frac{1}{2}C_{ijklmn}S_{mn})S_{kl}$$

Next, the expression in parenthesis could be defined as an elastic stiffness tensor:

(18) $$C_{ijkl}{}^{\prime }=C_{ijkl}+\frac{1}{2}C_{ijklmn}S_{mn},\;i,j,k,l=1,\;2,\;3$$

With this new definition, the linear second-order elastic stiffness tensor can be modified so that it is strain-dependent using the coefficients of nonlinear elasticity. Since working with tensors is not straight forward, Voigt notation should be used to convert tensorial format to matrix format. The same concept presented above is shown below in matrix format:

(19) $$T_{i}\left(X\right)=(C_{ij}+\frac{1}{2}C_{ijk}S_{k})S_{j}$$

$$C_{ij}{}^{\prime }=C_{ij}+\frac{1}{2}C_{ijk}S_{k}\;i,j,k=1,\;2,\;3,\;4,\;5,\;6$$

Now using Einstein notation and considering cubic symmetry for silicon structure, the equation above can be expanded to find all of the 36 components of the new modified stiffness matrix $C_{ij}{}^{\prime }$.

**Figure 4:**
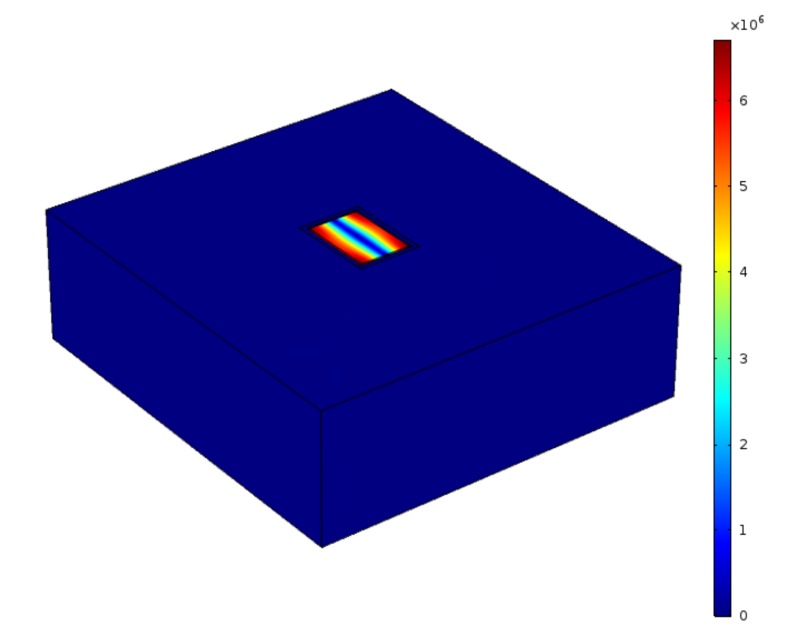
The structure developed in COMSOL for modeling the acceleration sensitivity.

The structure used in this work for modeling is shown in Figure 4, which includes the lateral extensional resonator connected to the substrate. It should be noted that in the proposed FEM model, only the silicon layer is considered. Utilizing such simplified model is justified as, firstly, the thickness of the AlN layer is 1/8th of the thickness of the silicon layer, and, secondly, the sputtered AlN used in these resonators is isotropic in *x*-*y* plane. Therefore, it is concluded that the orientation dependency of acceleration sensitivity in resonators is mainly dominated by their structural silicon body. 

To study vibration sensitivity using COMSOL, a prestressed analysis is performed on the discussed structure as follows: first, a stationary study is performed to obtain the final strain values due to the applied acceleration followed by a second step of eigenfrequency study to predict the modified natural resonance frequency while the initial strains calculated in the first step are applied to the structure. It should be noted that acceleration is applied as a body load equal to $\vec{F}=\rho \vec{a}$ (N/m^3^) to the whole structure including the frame, in which ρ is effective density. It is worth mentioning that COMSOL uses a perturbation model to calculate the eigenfrequencies of the pre-stressed system. Although it is possible to directly solve a set of differential equations to find the resonance frequency under acceleration but as described in [39]*,* it has been shown that calculating the first eigenvalue perturbation is considerably more efficient.

**Figure 5:**
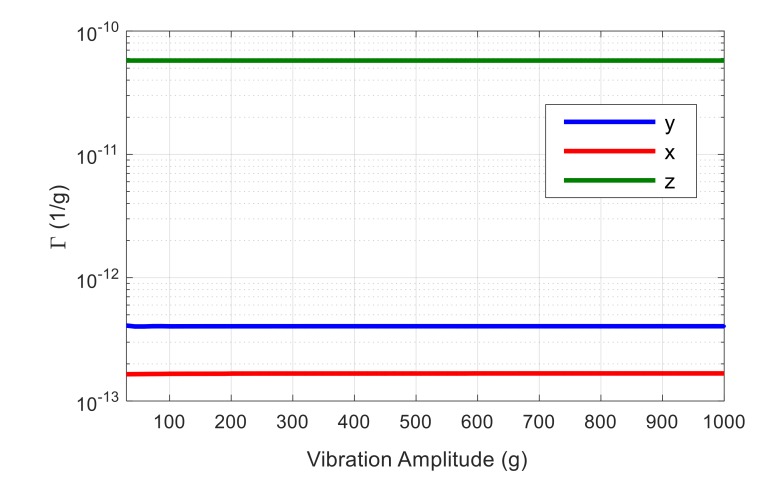
The simulated acceleration sensitivity in *x*, *y*, and *z* directions for a resonator with lateral extensional mode-shape aligned to <100> orientation.

**Figure 6:**
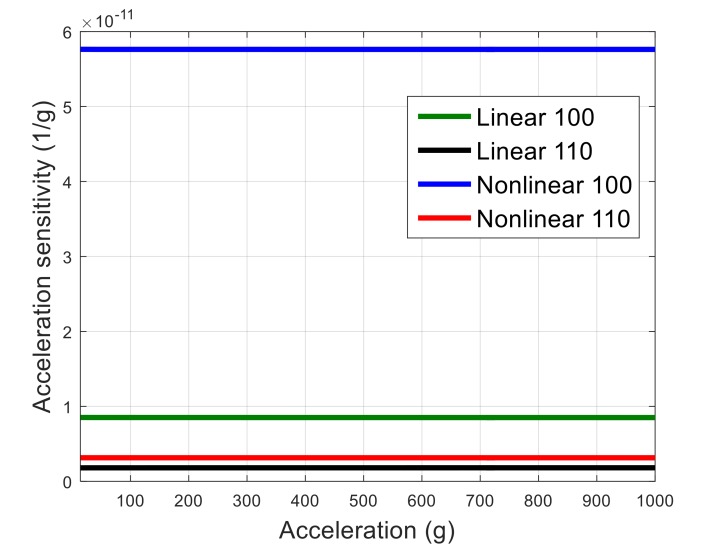
The simulated acceleration sensitivity considering linear/nonlinear elastic properties for both <100> and <110> crystalline orientations.

Acceleration sensitivity is calculated as normalized resonance frequency shift due to the applied acceleration using Equation (2). The resulted vibration sensitivity in *x*, *y*, and *z* direction as a function of acceleration amplitude is shown in Figure 5 for a resonator aligned to <100> orientation. As seen, the acceleration sensitivity in *z* direction is the dominant component of the Γvector, which is expected, as in this case vibration is applied normally to the resonator plane, introducing an out-of-plane bending moment with a relatively low effective stiffness in the structure. Hence, only acceleration sensitivity in *z* direction is demonstrated in the following figures for simplicity. Material nonlinearity for <100> orientation is defined in COMSOL, as explained before. In order to consider nonlinearity in <110> orientation, a 45° rotated coordinate system in the resonator plane is used. The simulated acceleration sensitivity for both resonators aligned to <100> and <110> orientations considering linear and nonlinear material properties is demonstrated in Figure 6.

As seen, acceleration sensitivity of the <100> resonator is larger than the <110> resonator, even for the case of linear material properties. One possible reason is that the Young’s modulus in <100> silicon is smaller and the Poisson ratio is larger than that of the <110> orientation [42]. Hence, when a load is applied in <100> direction normal to the plane of the resonator, larger bending results, and the in-plane deformation of the resonator would be larger for the resonator aligned to <100> plane compared with the resonator aligned to <110> plane. The resulting in-plane strain will directly affect the resonance frequency, as the frequency is a function of the resonator’s in-plane dimensions. Therefore, it appears that the dominant factor in the acceleration sensitivity of the resonator studied in this work is the Poisson ratio of silicon. However, it should be noted that material nonlinearity also affects the absolute value of acceleration sensitivity significantly. As seen, when material nonlinearity is considered, acceleration sensitivity is increased in both <100> and <110> cases. When silicon is defined as a linear material, the ratio of vibration sensitivity of resonator aligned with <100> to that aligned with <110> is 4.7, while this ratio increases to 18.5 when the material nonlinearity of silicon is considered. Therefore, material nonlinearity significantly affects both the value and the orientation dependency of the acceleration sensitivity.

It is worth mentioning that this simulation only considers the resonator and part of the substrate that is simplified compared to the experimental setups. In addition, elastic constants used in this simulation are for n-type silicon with doping concentration of 2 × 10^19^ cm^−3^ [43]*,* which is the closest available data to the doping level for the devices fabricated in this work (~5 × 10^19^ cm^−3^). Furthermore, defining material nonlinearity for other layers of the device including AlN may affect the absolute value of acceleration sensitivity. However, in this study, the goal is to investigate the effect of orientation on acceleration sensitivity of n-type highly doped silicon-based resonators and understand the effect of material nonlinearity on that. Thus, predicting the relative sensitivity in different orientations is the most important aim of this simulation, which can be achieved with this simplified model. 

**Figure 7:**
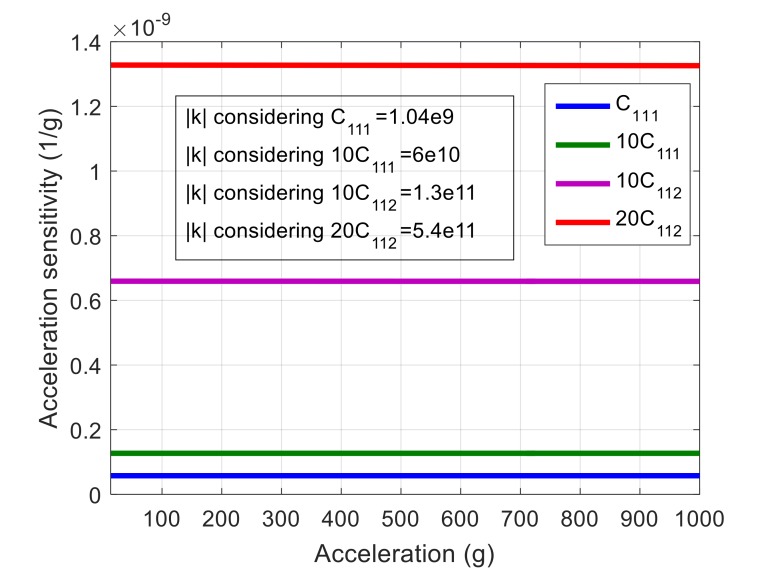
The acceleration sensitivity of <100> resonator using modified elastic constants, indicating the correlation between elastic nonlinearity and acceleration sensitivity.

From the above simulation, it is concluded that by increasing material nonlinearity, acceleration sensitivity will increase. To confirm this conclusion, nonlinear elastic coefficients of <100> silicon are intentionally modified so that a larger amplitude frequency, *A-f*, coefficient is obtained. This coefficient, *k*, is a measure of nonlinearity, which will be explained in more detail in Section 5. In order to calculate *k* as a function of nonlinear stiffness constants, first, the nonlinear Young’s modulus is calculated based on the closed form equations presented in [40]. Then, amplitude-frequency coefficient *k* is calculated using equations presented in [44] for lateral extensional mode-shape aligned with <100> orientation. The simulated acceleration sensitivity using the modified elastic constants is shown in Figure 7. As seen, by increasing *k* (i.e., nonlinearity), acceleration sensitivity also increases, which confirms the correlation between acceleration sensitivity and nonlinearity.

## 5. Measurements and Results

As explained in Section 1, we hypothesized that acceleration sensitivity of silicon-based MEMS resonators is orientation-dependent and affected by the elastic properties of the resonator material. In order to study this hypothesize, both nonlinearity and acceleration sensitivity of above resonators need to be measured.

### 5.1. Nonlinearity Measurements

Amplitude-frequency (*A-f*) coefficient *k* is a measure of nonlinearity in resonators. This coefficient determines the relation between normalized resonance frequency shift of the resonator and the amplitude of vibration (*x*) as presented by (20).

(20) $$f=f_{0}\left(1+kx^{2}\right)$$

We will use backbone curve plots obtained through the ringdown response measurement in order to evaluate nonlinearity of the resonators. A backbone curve is the plot of normalized frequency shift versus amplitude of vibration. In order to find the shift of resonance frequency in nonlinear regime, two methods can be used: forced or unforced oscillation. In former, the input excitation power is increased gradually, and the shift in resonance frequency is read using a network analyzer while the resonator is under forced excitation. However, in the latter, ringdown response of the resonator is measured, and by analyzing the decaying signal, shift in natural resonance frequency of the unforced device vibration can be calculated [45]. In this study, second method is used.

**Figure 8:**
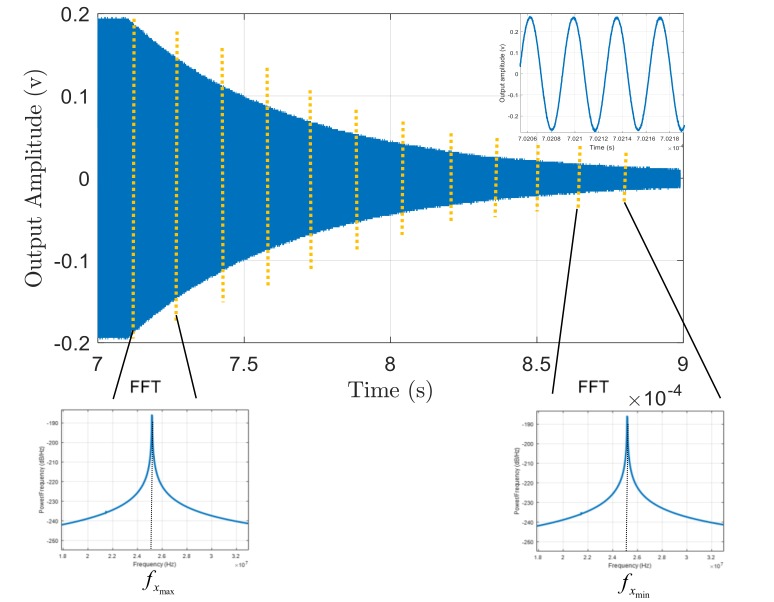
The ringdown response of the resonator and the FFT plots for different sections of the decaying signal to find the backbone curve. $f_{x_{max}}$ and $f_{x_{min}}$ are the resonance frequencies of the device corresponding to largest and smallest amplitudes of vibration, respectively.

Each resonator is forced by a large enough input that guarantees a detectable shift in resonance frequency. After exciting the resonator for a period of time, the input power is ceased, and the unforced ringdown response of the device is captured. The time domain behavior of the output voltage signal for one of the resonators used in this study captured by a digital oscilloscope is demonstrated in Figure 8.

**Figure 9:**
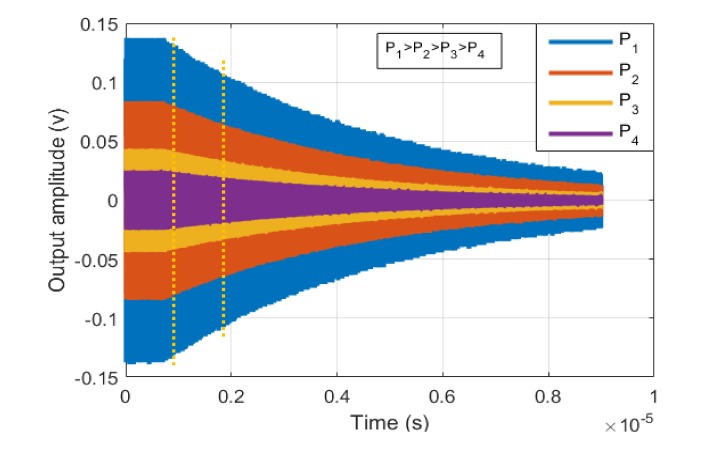
The ringdown response of the resonator for different input powers. The resonance frequencies corresponding to the highest and lowest power (P_1_ and P_4_) are $f_{x_{max}}$ and $f_{x_{min}}$, respectively.

The decaying signal is then spilt into several bins, and a fast Fourier transform (FFT) is used for the data in each bin to find the natural resonance frequency of the resonator corresponding to the varying output voltage (vibration amplitude) during the ringdown signal (Figure 8). Equivalently, instead of using FFT over one decaying signal, the resonator can be excited separately with different input powers. Then, for each input power FFT is taken over the first bin of decaying signal. Figure 9 shows the ringdown response for different input powers.

Now, in order to plot the backbone curve, the output voltage needs to be converted to the amplitude of vibration. For capacitive resonators, there already are closed form equations developed to perform this conversion [45]. However, for piezoelectric resonators such equations are not available in literature. So to find the amplitude of vibration for each input power, the S parameters of the resonators are collected for each case. Then, energy stored in resonator in each case is calculated using the method proposed in [46]. In this approach, the magnitude and phase of the input and output current and voltage of the resonator are calculated using a model developed in the advanced design system (ADS) software and the S-parameters of the device. The energy stored in resonator is then obtained using those calculated parameters [46]. This energy can be approximated by

(21) $$E=\frac{1}{2}k_{0}x^{2}$$

in which *x* is the amplitude of vibration and *k*_0_ is the linear spring stiffness constant of the resonator, which for lateral-extensional mode-shape can be calculated by [44]

(22) $$k_{0}=\frac{\pi^{2}EA}{2L}$$

in which *E* is the effective Young’s modulus, and *A* and *L* are the cross section area and length of the resonator, respectively. Using Equation (21), amplitude of vibration for each input power is calculated, and the backbone curves are plotted. The plot is then fitted with Equation (23) in order to find *A-f* coefficient *k*.

(23) $$\Delta f/f_{0}=kx^{2}$$

**Figure 10:**
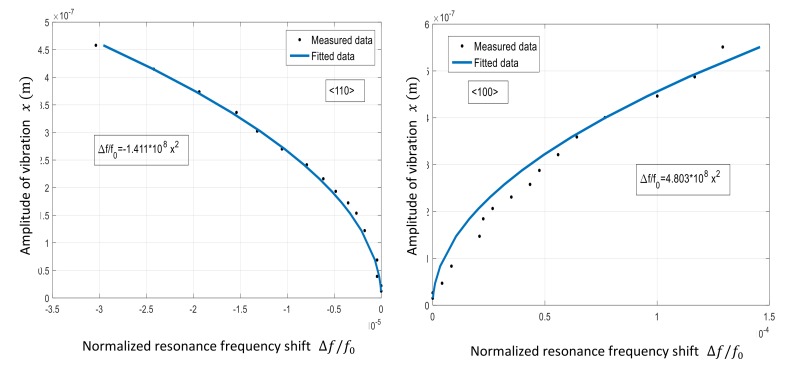
The backbone curve for <110> (**left**) and <100> (**right**) resonators and the associated amplitude-frequency (*k*) coefficients calculated based on the curves, indicating higher nonlinearity in the <100> resonator.

The backbone curve and the fitted plot for both <100> and <110> resonators in set 1 is shown in Figure 10. As seen, the absolute value of *k* for <100> resonator (4.803) is more than 3× larger than that calculated for the <110> resonator (1.411). This confirms a larger nonlinearity for the <100> resonator.

Hence, this resonator is expected to be more sensitive to acceleration, as simulation predicts more sensitivity by increasing nonlinearity.

### 5.2. Acceleration Sensitivity Measurements

**Figure 11:**
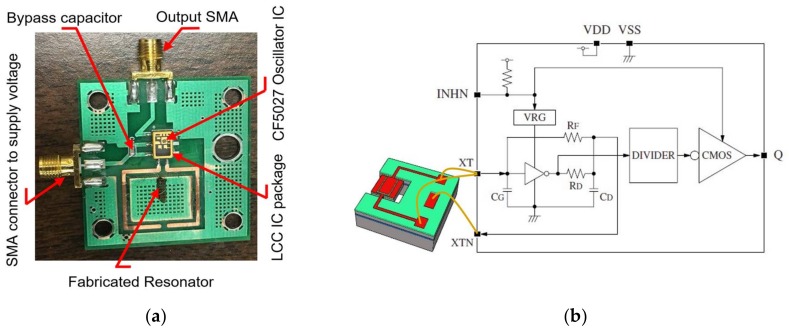
(**a**) The oscillator PCB containing the commercial oscillator IC (CF5027) and (**b**) the schematic of the oscillator IC connected to the TPoS resonator [47].

In order to measure acceleration sensitivity, the resonator must be employed in an oscillator circuit. To minimize the number of components, a commercial oscillator IC, CF5027 is used. The printed circuit board (PCB) and the schematic of the oscillator used in this study are shown in Figure 11a,b. The connections XT and XTN denoted on the IC are the amplifier input and output, into which the MEMS resonator should be connected. Details of the oscillator IC specifications can be found in the data sheet [47]. 

**Figure 12:**
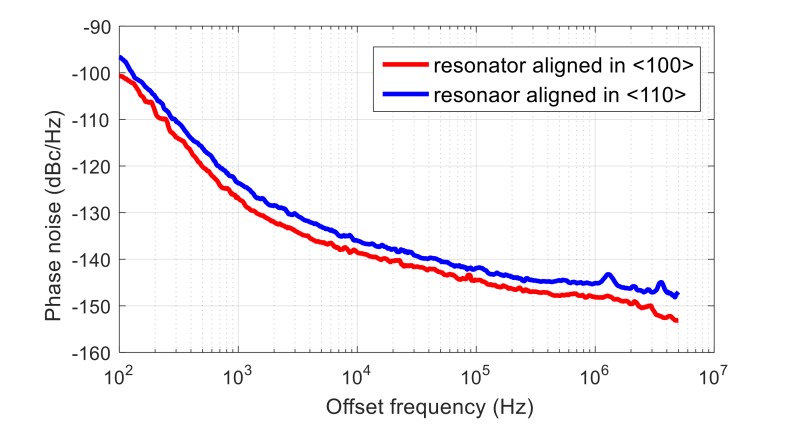
The phase noise performance of the oscillator circuits containing the <100> and <110> resonators.

The typical phase noise measured from the assembled oscillators is shown in Figure 12. As seen, both oscillators exhibit excellent phase noise performance, which enables accurate measurement of the acceleration sensitivity.

**Figure 13:**
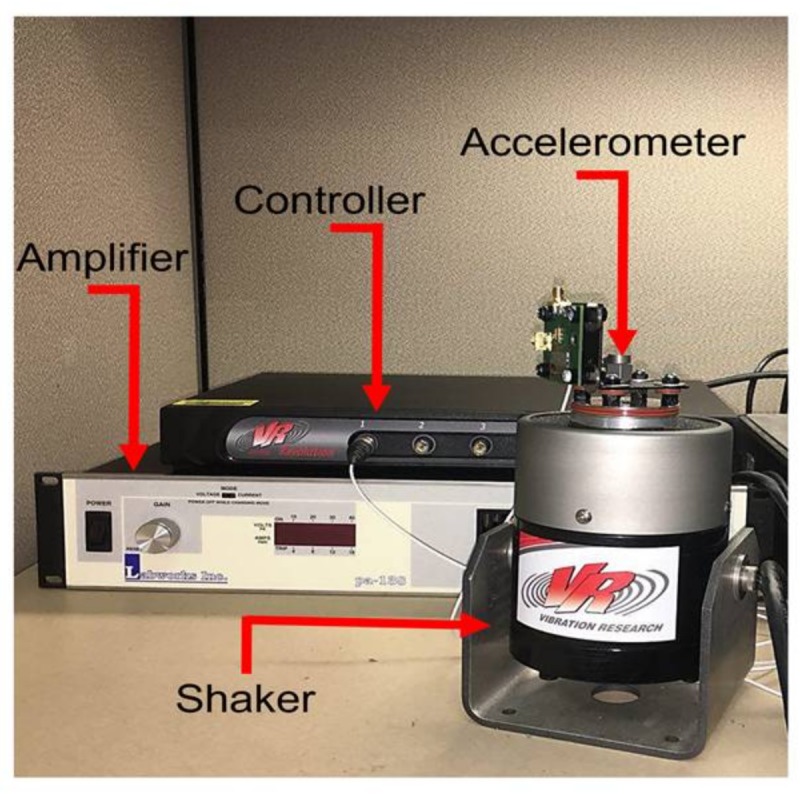
The acceleration-sensitivity measurement setup.

A closed loop magnetic shaker system from Vibration Research Group Inc. is used to simulate the actual vibration coming from environment (Figure 13). The acceleration applied to the board is measured by a DYTRAN 3055D1T accelerometer. The sensed acceleration is then fed back to the controller to be compared with the desired acceleration set by the operator. A control signal is afterward sent to the amplifier, which determines the power generated by the amplifier that feeds the magnetic motor.

**Figure 14:**
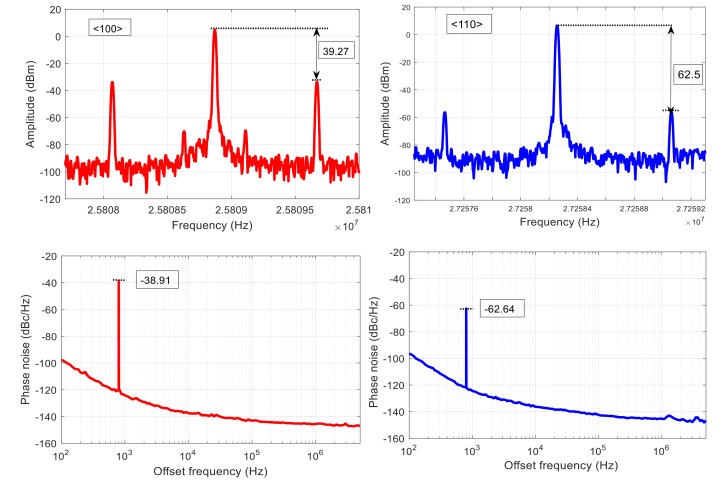
The oscillator output spectrum and the phase noise under 14 g, 800 Hz vibration for set 1 of resonators. Larger amplitude of the sidebands that appeared at 800 Hz offset frequency from the carrier in the output frequency spectrum of the oscillator with <100> resonator shows higher acceleration sensitivity of this device. More theoretical details are provided in Section 2.

The phase noise and the output spectrum of the two resonators aligned with <100> and <110> orientations are shown in Figure 14 when a 14 g, 800 Hz sinusoidal acceleration is applied in *z* direction. As seen, two sidebands appear at an offset frequency of 800 Hz from the carrier in the oscillator output spectrum for both resonators, as expected from theoretical explanations provided in Section 2. The amplitude of these sidebands is proportional to the acceleration sensitivity of resonator. Hence, it is obvious that resonator aligned to <100> plane is significantly more sensitive to the acceleration compared with the one aligned to the <110> plane. Acceleration sensitivity of these devices is 3.1 × 10^−9^ and 4.8 × 10^−8^ 1/g for resonators aligned with <110> and <100> orientations, respectively. Spurious signals will also appear on the phase noise plot at an offset frequency equal to the vibration frequency. In fact, the sidebands on the output spectrum are considered as noise, and hence the corresponding spurs on the phase noise are expected. It should be noted that the amplitude of the spurs on the phase noise is almost equal to the difference between amplitude of the carrier and sidebands in the spectrum, which corresponds to how the phase noise is calculated.

It is worth mentioning that, in this study, the phase noise and frequency spectrum measurements are done with a Rohde & Schwarz signal source analyzer. However, one can configure a platform to do the same measurement, such as those presented in [48,49].

**Figure 15:**
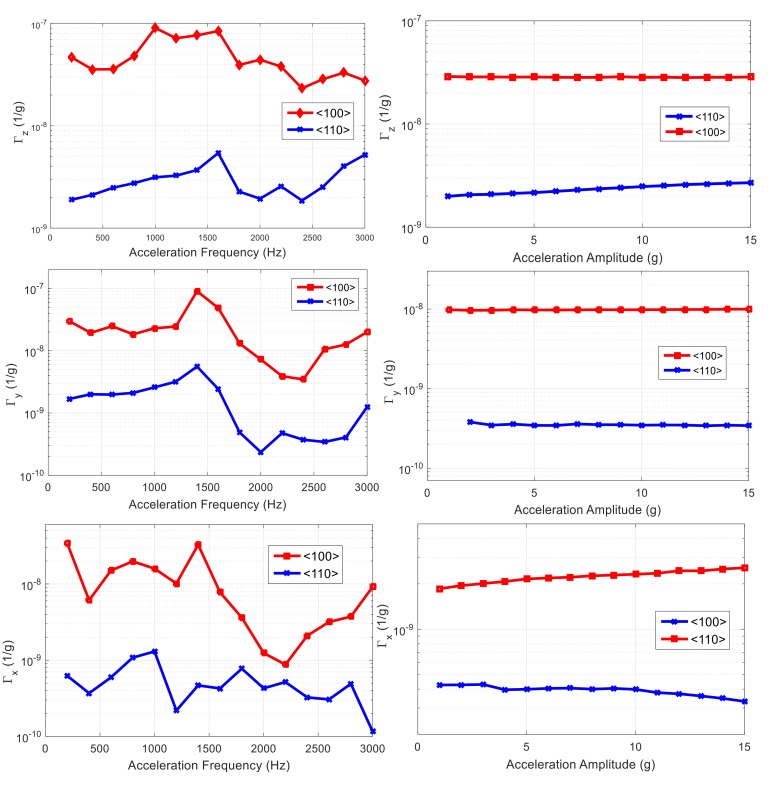
Acceleration sensitivity in *x*, *y*, *z* direction vs. acceleration frequency (**left**) and magnitude (**right**) for set 1 of resonators.

Acceleration sensitivity of all resonators has been measured by sweeping both acceleration amplitude and frequency. The results for first set of resonators are shown in Figure 15. By averaging total acceleration sensitivity over vibration frequency range of 0–3 kHz, ~5.66 × 10^−8^ and ~3.66 × 10^−9^ are obtained for resonators aligned to <100> and <110> orientations, respectively.

It is worth mentioning that, contrary to the measurement, the acceleration sensitivity is not expected to vary with acceleration frequency. This is because the induced strain in the resonant structure should not be a function of the acceleration frequency, as no resonance modes of the device are within the range of applied frequency. However, the acceleration is not directly applied to the device, and, rather, it is being applied to a board to which the resonator die is attached. It is suspected that there are numerous resonance modes for the entirety of the board and the components it contains including the wirebonds, cables, and connectors. Therefore, the effective force applied to the resonator will end up varying with the frequency. In addition, other sources of acceleration sensitivity such as a shift in the phase can play a role that is frequency-dependent.

The same measurement has been repeated for the second set of devices, and the same trend as for the first is obtained, confirming our hypothesis on orientation-dependency of acceleration sensitivity. Acceleration sensitivity of both sets of resonators for a 7 g, 2800 Hz sinusoidal vibration is reported in Table 1.

## 6. Conclusions

The acceleration sensitivity of n-type, highly-doped, silicon-based extensional resonators aligned with different crystalline orientations is studied. Experimental results suggest that a resonator aligned with the <110> plane direction is much less sensitive to applied acceleration compared with a similar resonator aligned with <100> plane. A finite element model is presented to simulate the acceleration sensitivity of the resonators used in this study. Simulation results also confirm less sensitivity for the <110> resonator. In addition, it was shown that material nonlinearity is an important factor affecting the acceleration sensitivity of these type of resonators.

## Figures and Tables

**Table 1:** Acceleration sensitivity for both sets of devices for a 7 g, 2800 Hz sinusoidal vibration.

	Orientation	Г*_x_*	Г*_y_*	Г*_z_*	Г_total_
**Set 1**	<100>	3.73 × 10^−9^	1.2 × 10^−8^	3.3 × 10^−8^	3.6 × 10^−8^
	<110>	4.9 × 10^−10^	4.1 × 10^−10^	4 × 10^−9^	4.1 × 10^−9^
**Set 2**	<100>	8.5 × 10^−9^	2.5 × 10^−8^	8.1 × 10^−8^	8.5 × 10^−8^
	<110>	3.1 × 10^−10^	6.4 × 10^−9^	4.8 × 10^−9^	8 × 10^−9^
